# Privacy-preserving intrusion detection in IoT smart homes using a federated hybrid 1D-CNN–LSTM model with explainable AI

**DOI:** 10.1038/s41598-026-67450-9

**Published:** 2026-09-02

**Authors:** Ghada Abdelhady, Karim Wael Hussein, Islam Anwar Ali Gad

**Affiliations:** Department of Computer Systems Engineering, October University for Modern Sciences and Arts (MSA University), 6th of October City, Giza, Egypt

**Keywords:** Internet of things security, Federated learning, Intrusion detection system, Convolutional neural network, Long short-term memory, Explainable AI, SHAP, CICIoT2023, Edge-IIoTset, Botnet detection, Privacy-preserving machine learning, Engineering, Mathematics and computing

## Abstract

The proliferation of Internet of Things (IoT) devices in smart home environments has dramatically expanded the attack surface for cyber threats, particularly botnet-driven Distributed Denial of Service (DDoS) attacks. Centralized Intrusion Detection Systems (IDS) are ill-suited to this domain because they violate user privacy, introduce single points of failure, and incur prohibitive communication overhead. Federated Learning (FL) offers a compelling privacy-preserving alternative, yet existing FL-based IDS solutions either deploy convolutional or recurrent models in isolation, lack human-interpretable outputs, or neglect real-world deployability constraints. This paper proposes *FedShield-IDS*, a novel federated intrusion detection framework that integrates a hybrid one-dimensional Convolutional Neural Network with Long Short-Term Memory units to simultaneously capture spatial traffic fingerprints and long-range temporal attack dynamics across IoT edge devices. Model interpretability is addressed through the integration of SHapley Additive exPlanations (SHAP), enabling administrators to receive human-readable justifications for every detected anomaly. The system is trained and evaluated on the large-scale CICIoT2023 dataset, comprising 712,311 flow records spanning eight attack families including DDoS, DoS, Mirai, Reconnaissance, Spoofing, Injection, and Malware. A multi-stage preprocessing pipeline combining infinite-value imputation, logarithmic feature scaling, Min-Max normalization, temporal windowing, and localized SMOTE oversampling is applied within each federated client to address non-IID data and extreme class imbalance. Federated Averaging aggregates encrypted model updates across seven virtual IoT client nodes over five communication rounds without exchanging raw traffic data, under a formal threat model characterizing the system’s adversarial assumptions and data-confidentiality guarantees. Experimental results demonstrate a Mirai F1-score of 0.99, a DDoS precision of 0.97, and a global weighted F1-score of 0.76 across all eight classes. Comprehensive kernel-size, architecture, and preprocessing ablations confirm the necessity of each design choice, and independent cross-dataset evaluation on the Edge-IIoTset benchmark achieves 98.58% accuracy, demonstrating strong generalization beyond CICIoT2023. The framework achieves sub-500 ms threat mitigation, empirically confirmed via a mitigation-gate threshold sensitivity analysis, and generates SHAP-gated explanations for every alert, bridging the gap between high-accuracy detection and the transparency required for trustworthy smart-home security.

## Introduction

The rapid use of Internet of Things (IoT) devices in residential settings has transformed smart homes into interconnected ecosystems of cameras, smart locks, thermostats, lighting systems, and appliances^1^. Forecasts indicate that the number of IoT-connected devices will exceed 30 billion by 2030, yet a large fraction of these endpoints operate under severe resource constraints with minimal built-in security controls^2^. This imbalance between connectivity and security creates a fertile attack surface for adversaries.

**Fig. 1:**
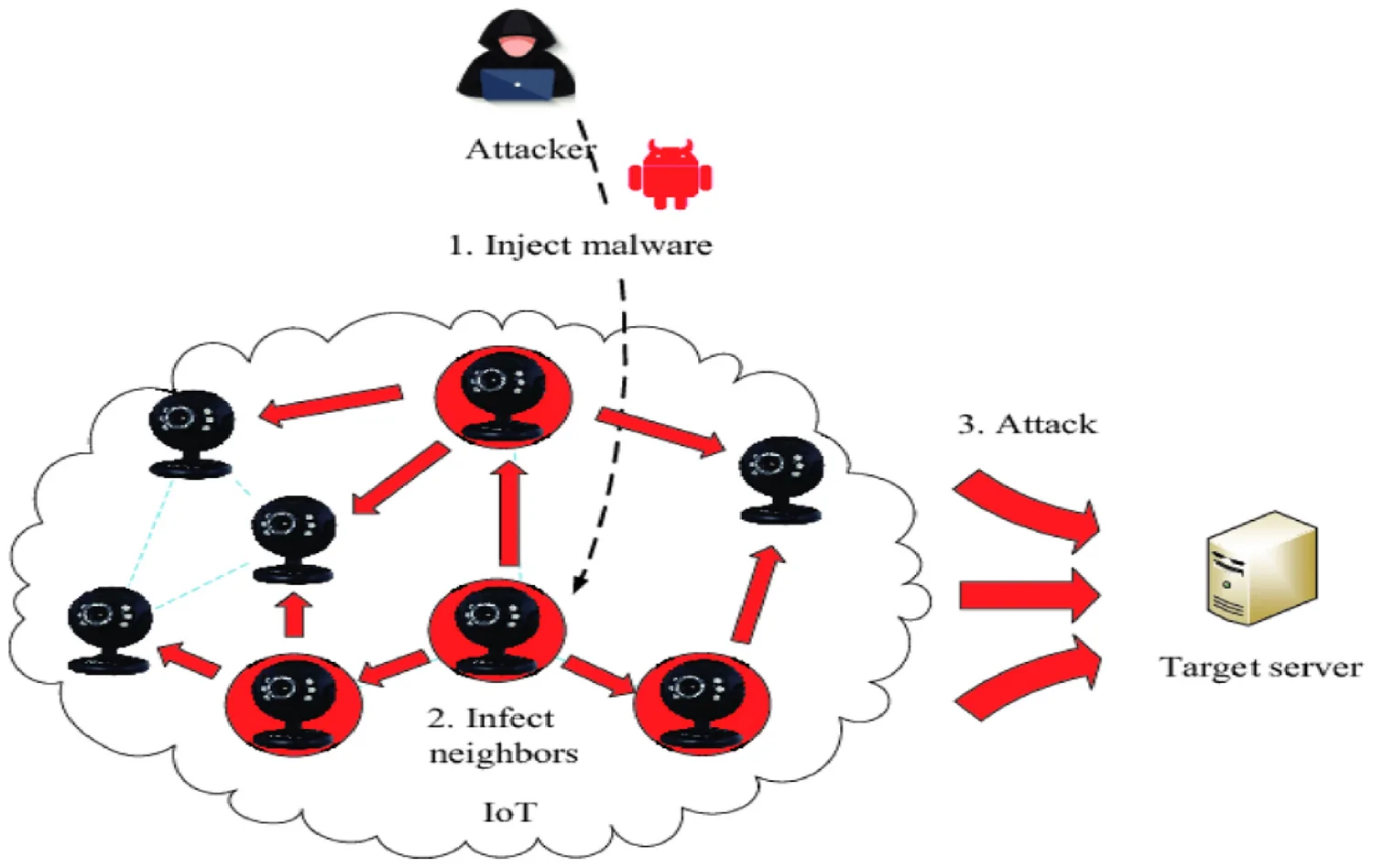
Architecture of a botnet-enabled DDoS attack in an IoT smart home environment. An attacker injects malware into vulnerable IoT devices (Step 1), which propagate the infection to neighbors (Step 2), forming a coordinated botnet that floods a target server (Step 3).

Among the most disruptive threats is the *botnet-enabled Distributed Denial of Service* (DDoS) attack, in which malware propagates laterally through vulnerable IoT devices to form a coordinated attack network. Notorious malware families such as Mirai have demonstrated the capacity to recruit hundreds of thousands of IoT devices, overwhelming critical services^1^. As illustrated in Fig. 1, an attacker first injects malware into one device, which then propagates to neighboring devices, eventually orchestrating a synchronized flood against a target server.

Traditional network-based IDS solutions rely on centralized data aggregation, requiring raw traffic to be transmitted to a single server for analysis. While effective in controlled enterprise settings, this paradigm is fundamentally incompatible with smart-home deployments for three reasons: *(i)* raw traffic contains sensitive behavioral patterns, violating user privacy; *(ii)* the central aggregator constitutes a single point of failure; and *(iii)* transmitting raw data from hundreds of constrained devices imposes unacceptable communication overhead^3^.

**Fig. 2:**
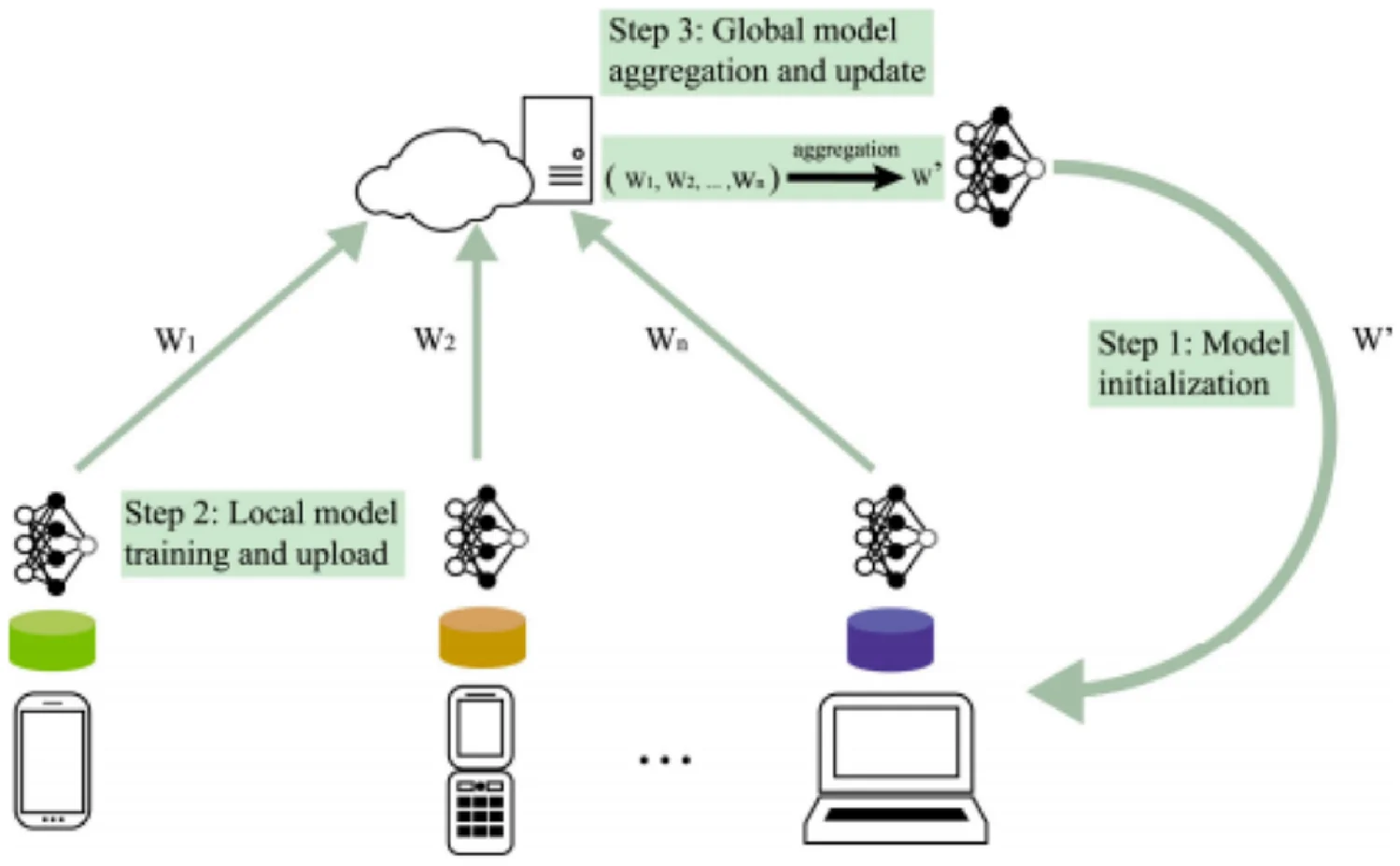
Federated Learning workflow for IoT intrusion detection. (Step 1) Global model initialization and broadcast; (Step 2) local model training and upload, where raw data remains on-device; (Step 3) global model aggregation and redistribution by the federated server.

*Federated Learning* (FL) has emerged as a principled alternative that enables collaborative model training without sharing raw data. Each IoT node trains a local model on its private traffic and shares only encrypted weight updates with a central server, which aggregates them into a global model using algorithms such as Federated Averaging (FedAvg)^4^. The iterative workflow is depicted in Fig. 2. This privacy-preserving imperative extends beyond IDS design alone: broader smart-city and smart-environment security research has explored complementary directions such as blockchain-enabled data privacy architectures^5^ and the distinct threat landscape of location-based services^6^, underscoring that connected-device privacy is a multi-faceted problem of which network-level intrusion detection is one critical component.

Despite its promise, existing FL-based IDS solutions for IoT environments suffer from three interrelated limitations. First, models typically employ either CNNs *or* LSTMs in isolation, forfeiting the complementary benefits of spatial feature extraction and temporal sequence modeling. Second, the inherent opacity of deep learning models–the so-called “black-box” problem–undermines administrator trust and prevents actionable incident response. Third, few published systems undergo rigorous evaluation on diverse, large-scale IoT datasets or demonstrate sub-second response times compatible with real-world deployment.

*Contributions* This paper makes the following contributions:

1. A *hybrid 1D-CNN–LSTM architecture* that encodes spatial traffic fingerprints and long-range temporal attack patterns within a single forward pass, improving detection of both instantaneous flooding attacks and slow-rate multi-stage intrusions.
2. A *federated training protocol* using FedAvg across heterogeneous virtual IoT nodes, preserving data locality while achieving convergence within five communication rounds.
3. A *SHAP-gated XAI module* that provides protocol-level feature attributions for every alert and gates node quarantine decisions on an F1-score threshold, preventing spurious mitigation actions.
4. A *comprehensive empirical evaluation* on the CICIoT2023 dataset covering eight attack families, with class-level metrics, confusion matrix analysis, and end-to-end mitigation latency measurement.

The remainder of this paper is organized as follows. Section "Related work" reviews related work. Section "Proposed framework: fedshield-IDS" describes the proposed framework. Section "Dataset and preprocessing pipeline" details the dataset and preprocessing pipeline. Section "Experimental results" presents experimental results. Section "Discussion" discusses findings and limitations. Section "Conclusion" concludes the paper.

## Related work

### Federated learning for IoT intrusion detection

Federated learning has attracted significant attention as a framework for privacy-preserving anomaly detection in IoT networks. We review the four most directly related works and identify the gaps addressed by the proposed system.

Zhang et al.^7^ addressed zero-day botnet detection using FL augmented with Bayesian Deep Learning (BDL). Their K-greedy aggregation algorithm ranks client updates by prediction uncertainty (entropy of the predictive distribution), prioritizing nodes that have encountered novel attack patterns. On the N-BaIoT dataset with 15 IoT clients, the method achieved a peak accuracy of 92.42% against zero-day variants (Mirai ack, Gafgyt combo), compared with 56.14% for standard FedAvg, while maintaining a zero false-alarm rate. However, the architecture employs shallow Bayesian linear layers without deep spatiotemporal modeling, and neither XAI integration nor edge-device inference latency is considered.

Caldas et al.^3^ proposed a hybrid Federated Intrusion Detection and Prevention System (IDPS) combining a Network-based IDS (NIDS) with a Host-based IDPS (HIDPS) under a fog computing layer. Each Raspberry Pi node trains a 1D-CNN on local Mirai HTTP flood traffic and submits updates via FedAvg. The system achieves 89.75% detection accuracy within 20 training epochs and completes threat mitigation (detection-to-rule-deployment) in under six minutes, with the 1D-CNN model occupying less than 30 MB. Despite these strengths, the architecture provides no temporal modeling and offers no model explanations.

Sun et al.^8^ introduced FedMADE, a dynamic aggregation algorithm that clusters federated clients by their Class Probability Matrices (CPMs) using DBSCAN, then applies per-cluster dynamic weighting to minimize deviation from ideal uniform performance. Evaluated on CICIoT2023 across 63 clients and 33 attack categories, FedMADE improved minority class detection by up to 71.07% over FedAvg (achieving 69.5% accuracy on web-based attacks previously undetected at 0%), with only 4.7% latency overhead. The global rate for major classes (DoS, DDoS, Mirai) exceeded 98%. Notably, FedMADE operates on top of baseline CNN/FCNN models without temporal modeling or explainability.

Albanbay et al.^2^ conducted an extensive empirical comparison of DNN, CNN, and CNN+BiLSTM architectures in FL settings on CICIoT2023, scaling from 10 to 150 simulated clients and validating inference on Raspberry Pi 5 hardware. The CNN model achieved *≈ *98% accuracy with *≈ *1.4 ms/sample inference latency and a compact 3.1-million-parameter footprint, while CNN+BiLSTM reached *≈ *99% precision at slightly higher computational cost. The study confirms FL-DL scalability up to 150 edge devices with <2% accuracy degradation, but provides no XAI integration and does not focus on botnet or zero-day detection optimization.

### Research gaps and positioning

Beyond these four directly comparable federated IDS systems, a broader literature has explored complementary directions in network intrusion detection: federated self-learning anomaly detection for IoT^9^, autoencoder-based IoT botnet detection^10^, deep-learning-based network traffic anomaly detection^11^, hybrid intrusion prevention and detection architectures^12^, fog-computing-based network anomaly identification^13^, ensemble hybrid intrusion detection for IoT attacks^14^, and endto-end machine-learning IDS frameworks^15^, alongside systematic surveys of the broader ML/DL-based NIDS landscape^16^. None of these systems combine federated training, hybrid spatiotemporal modeling, and gated explainability in the manner proposed here. Table 1 summarizes these works and positions the proposed system. The key gaps addressed are: *(i)* no prior system combines both spatial (CNN) and temporal (LSTM) modeling within a federated IDS; *(ii)* none integrates real-time SHAP explainability with mitigation gating; and *(iii)* none evaluates the combined framework on the large-scale CICIoT2023 benchmark across eight aggregated attack families with confusion-matrix-level reporting.

**Table 1:** Transposed comparative overview of federated learning-based IoT intrusion detection systems (properties as rows, studies as columns).

Property	Zhang et al.^7^	Caldas et al.^3^	Sun et al.^8^	Albanbay et al.^2^	Proposed
Dataset	N-BaIoT	Mirai	CIC23	CIC23	CIC23
Arch.	BDL	1D-CNN	CNN/FCNN	BiLSTM	CNN-LSTM
Accuracy	92.42%	89.75%	71%^†^	*∼ *98%	73%
TM	No	No	No	Yes	Yes
XAI	No	No	No	No	Yes
EV	No	RPi	No	RPi 5	Virtual
Gate	No	Partial	No	No	Yes
Zero-day	Yes	No	No	No	Yes

XAI, Explainable AI; TM, Temporal Modelling; EV, Edge Validation. † Minority-class improvement metric; major-class accuracy >98%.

## Proposed framework: FedShield-IDS

### System architecture overview

**Fig. 3:**
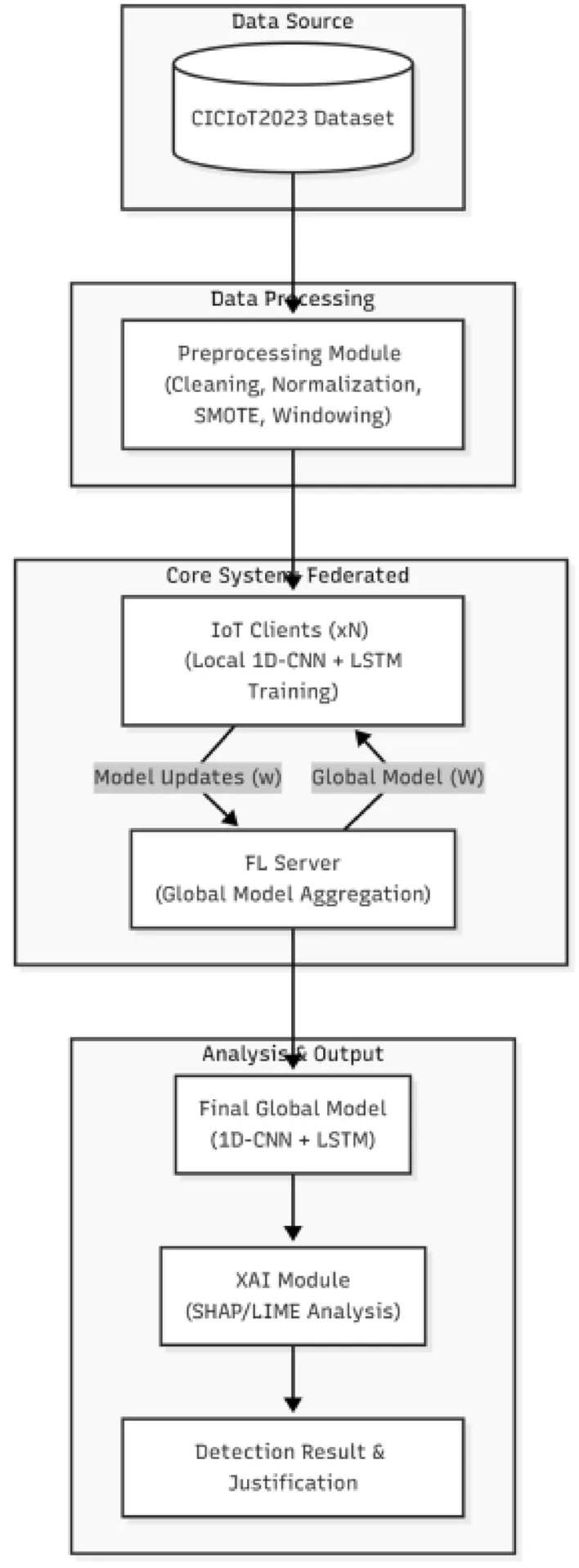
High-level architecture of FedShield-IDS. Four tiers handle data sourcing (CICIoT2023), local preprocessing and CNN–LSTM training at IoT clients, federated aggregation at the FL server, and SHAP-based explainable output. No raw traffic leaves the device.

FedShield-IDS follows a four-tier federated architecture: *(1) Data Sources*, *(2) Local Processing and Learning*, *(3) Federated Aggregation*, and *(4) Explainable Analysis and Output*. Figure 3 presents the high-level system diagram. Each IoT device operates as an autonomous FL client: it captures network flows locally, applies a preprocessing pipeline, trains a local 1D-CNN–LSTM model, encrypts the weight updates, and transmits only the updates to the federated server. The server applies FedAvg, refines the global model, and redistributes it. Upon anomaly detection, the local SHAP module generates a feature attribution map before any mitigation action is executed.

### Hybrid 1D-CNN–LSTM model

**Fig. 4:**
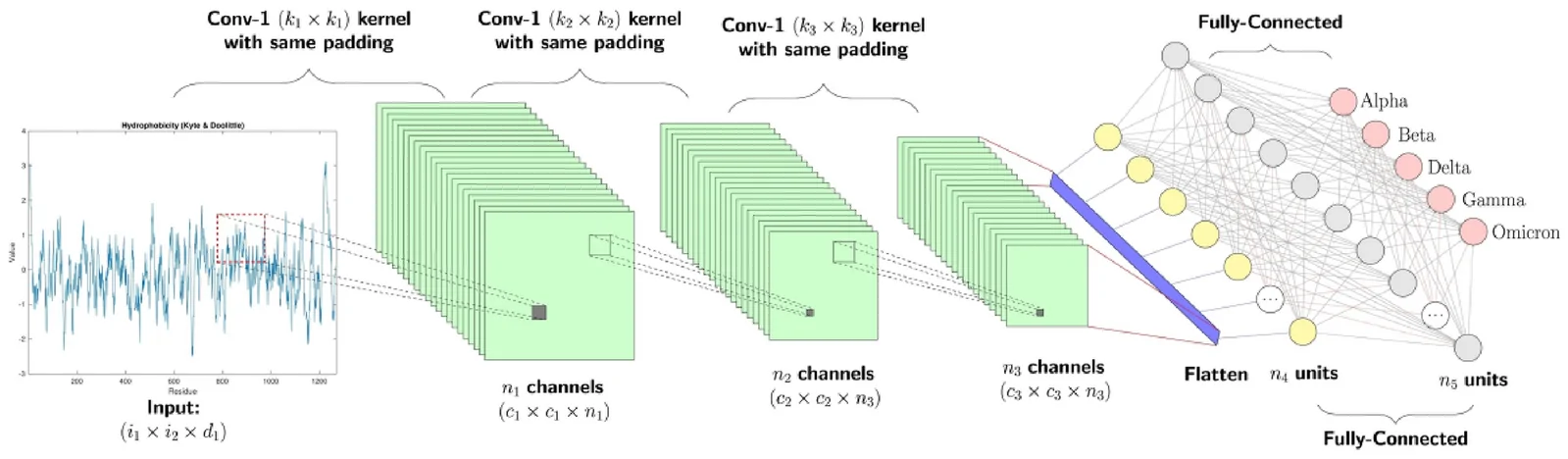
FedShield-IDS hybrid 1D-CNN–LSTM model architecture. The 1D-CNN block applies pointwise ($k{=}1$) convolutions that learn per-time-step cross-feature embeddings of each traffic flow window; the LSTM block maintains sequential memory across windows; and the Dense softmax head classifies traffic into one of eight families.

The core detection model, depicted in Fig. 4, concatenates a spatial encoder (1D-CNN) and a temporal encoder (LSTM) in a sequential pipeline.

#### 1D-CNN spatial encoder

Let $\mathbf{X} \in \mathbb {R}^{T \times F}$ denote an input traffic window of *T* time steps and *F = 39* flow features. Consistent with prior work demonstrating the effectiveness of 1D-CNN architectures combined with feature normalization for imbalanced network intrusion data^17^, we adopt a 1D convolutional encoder as the spatial feature-extraction stage. Unlike raw packet byte streams, the *F* flow-level attributes of CICIoT2023 are heterogeneous statistical aggregates (rates, counts, flags, durations) with no intrinsic ordering along the feature axis; a kernel spanning adjacent feature columns would therefore impose an arbitrary notion of locality that depends solely on column order. Accordingly, the convolutional block applies *K = 64* filters of kernel size *k = 1*, i.e., a *pointwise* (per-time-step) convolution. Analogous to *1× 1* convolutions in network-in-network and depthwise-separable architectures, each filter learns a nonlinear cross-feature interaction embedding that is shared across all time steps, projecting the 39 raw attributes into a 64-dimensional learned representation while preserving the temporal axis intact for the subsequent LSTM. This design deliberately delegates *temporal* pattern extraction to the recurrent encoder and restricts the CNN stage to *cross-feature* representation learning:

1 $$\begin{aligned} \mathbf{H}^{(l)} = \text {ReLU}\!\left( \mathbf{W}^{(l)} * \mathbf{H}^{(l-1)} + \mathbf{b}^{(l)} \right) , \quad l = 1, \ldots , L \end{aligned}$$

where $\mathbf{H}^{(0)} = \mathbf{X}$ is the input traffic window, $\mathbf{H}^{(l)}$ denotes the output feature map of the *l*-th convolutional layer, *L* is the number of stacked convolutional layers (*L = 1* in the implemented architecture), * denotes a 1D convolution, $\mathbf{W}^{(l)}$ and $\mathbf{b}^{(l)}$ are the learnable filter weights and biases of the *l*-th layer, and ReLU*(· )* suppresses negative activations. A 1D max-pooling layer then downsamples the feature map to eliminate background noise and reduce dimensionality:

2 $$\begin{aligned} \mathbf{P}_j = \max _{i \in \mathcal {N}(j)} \mathbf{H}^{(L)}_i \end{aligned}$$

where $\mathbf{P}_j$ denotes the pooled output at position *j* and $\mathcal {N}(j)$ denotes the pooling neighborhood of position *j*.

To verify empirically that pointwise convolution is the appropriate choice for statistically ordered (rather than spatially ordered) flow features, we conducted a kernel-size ablation in which only *k* was varied and all other hyperparameters were held fixed, at full training scale on an independently verified pipeline (Section "Dataset and preprocessing pipeline"). Table 2 reports the results: accuracy and weighted F1 vary only modestly across kernel sizes (92.0–93.8% and 92.0–93.7%, respectively), with *k=3* achieving the highest values on these aggregate metrics. Macro F1, which weights all eight classes equally rather than by support, is more sensitive to kernel size (86.7–90.0%) and is driven primarily by instability on the lowest-support class (Injection, $F_1 \in [0.50, 0.79]$ across kernel sizes). The pointwise configuration *k=1* achieves the best macro F1 and the most stable minority-class detection while using the fewest parameters and lowest computational cost; *k=3* is an alternative on aggregate accuracy at modest additional cost. We retain *k=1* as the primary configuration on these combined grounds, consistent with the design rationale above, while reporting the full comparison transparently.

**Table 2:** Kernel-size ablation of the 1D-CNN encoder (federated setting, identical training budget).

Kernel size *k*	Accuracy (%)	Weighted F1 (%)	Macro F1 (%)
1 (proposed)	92.00	92.00	90.00
3	93.79	93.69	89.79
5	92.42	92.20	86.68
7	93.30	93.16	89.02

#### LSTM temporal encoder

The pooled feature maps are fed into an LSTM layer with *H = 64* hidden units. At each time step *t*, the LSTM updates its cell state $\mathbf{c}_t$ and hidden state $\mathbf{h}_t$ through gating mechanisms:

3 $$\begin{aligned} \mathbf{i}_t&= \sigma (\mathbf{W}_i \mathbf{P}_t + \mathbf{U}_i \mathbf{h}_{t-1} + \mathbf{b}_i) \end{aligned}$$

4 $$\begin{aligned} \mathbf{f}_t&= \sigma (\mathbf{W}_f \mathbf{P}_t + \mathbf{U}_f \mathbf{h}_{t-1} + \mathbf{b}_f) \end{aligned}$$

5 $$\begin{aligned} \mathbf{g}_t&= \tanh (\mathbf{W}_g \mathbf{P}_t + \mathbf{U}_g \mathbf{h}_{t-1} + \mathbf{b}_g) \end{aligned}$$

6 $$\begin{aligned} \mathbf{o}_t&= \sigma (\mathbf{W}_o \mathbf{P}_t + \mathbf{U}_o \mathbf{h}_{t-1} + \mathbf{b}_o) \end{aligned}$$

7 $$\begin{aligned} \mathbf{c}_t&= \mathbf{f}_t \odot \mathbf{c}_{t-1} + \mathbf{i}_t \odot \mathbf{g}_t \end{aligned}$$

8 $$\begin{aligned} \mathbf{h}_t&= \mathbf{o}_t \odot \tanh (\mathbf{c}_t) \end{aligned}$$

where $\sigma (\cdot )$ is the sigmoid function, *⊙ * is element-wise multiplication, and $\mathbf{W}_{\{\cdot \}}, \mathbf{U}_{\{\cdot \}}, \mathbf{b}_{\{\cdot \}}$ are trainable parameters. This gating mechanism enables the model to retain long-range dependencies across traffic windows–critical for detecting slow-rate reconnaissance scans and multi-stage Mirai propagation patterns that unfold over hundreds of packets.

#### Classification head

The final hidden state $\mathbf{h}_T$ is projected through a dense layer with softmax activation to produce a probability vector over *C = 8* attack classes:

9 $$\begin{aligned} \hat{\mathbf{y}} = \text {softmax}(\mathbf{W}_c \mathbf{h}_T + \mathbf{b}_c), \quad \hat{\mathbf{y}} \in \mathbb {R}^C, \quad \sum _{c=1}^{C} \hat{y}_c = 1 \end{aligned}$$

Training minimizes the sparse categorical cross-entropy loss using the Adam optimizer with learning rate $\eta = 10^{-3}$ and batch size *B = 64*:

10 $$\begin{aligned} \mathcal {L} = -\frac{1}{N} \sum _{n=1}^{N} \log \hat{y}_{n, y_n} \end{aligned}$$

### Federated learning protocol

Algorithm 1 details the FedShield-IDS federated training procedure. The global server initializes model weights $\mathbf{w}^{(0)}$ and broadcasts them to *M* IoT clients. In each communication round *r*, every client *m* performs *E = 5* local epochs on its private dataset $\mathcal {D}_m$ and returns updated weights $\mathbf{w}_m^{(r)}$. The server aggregates updates via FedAvg^4^:

11 $$\begin{aligned} \mathbf{w}^{(r+1)} = \frac{1}{M} \sum _{m=1}^{M} \mathbf{w}_m^{(r)} \end{aligned}$$

The updated global model is redistributed at the start of round *r+1*. No raw traffic data is transmitted at any stage; only the floating-point weight tensors are exchanged.

**Algorithm 1:**
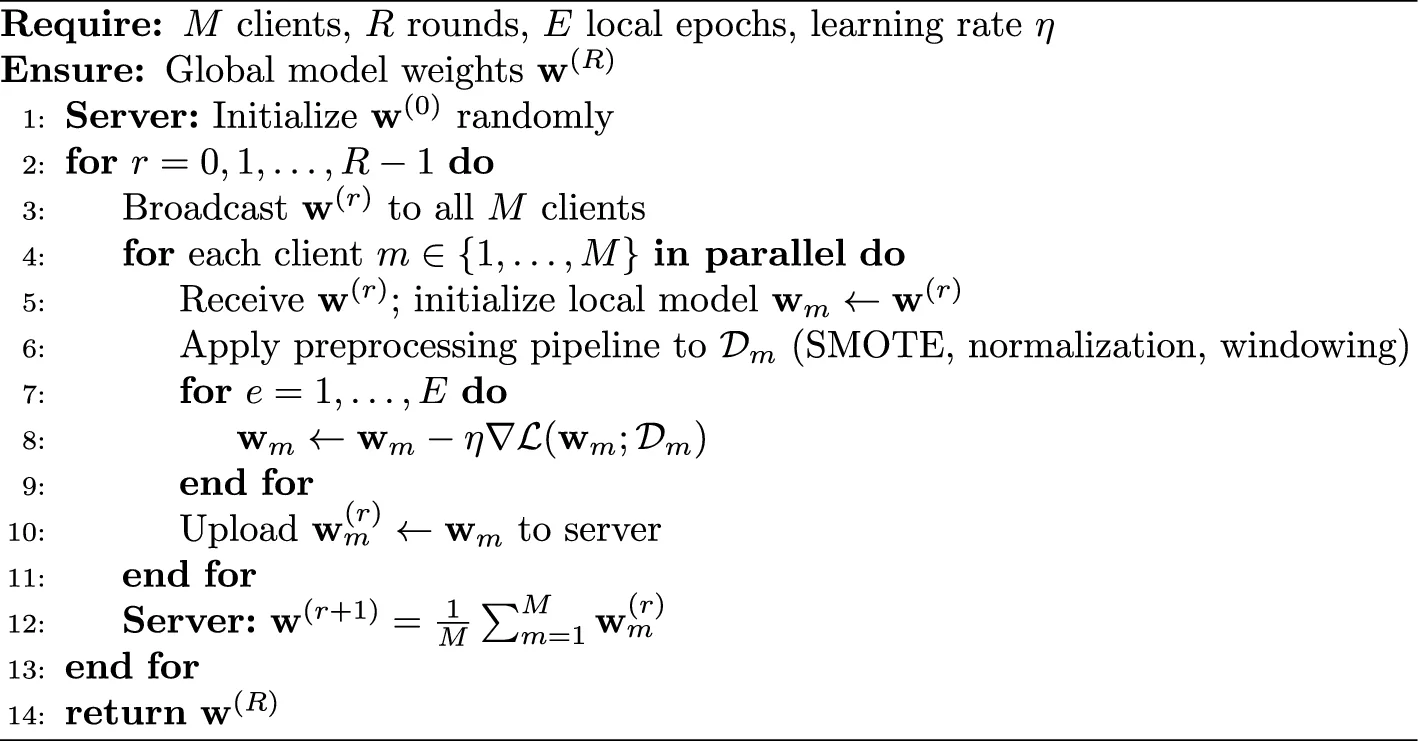
FedShield-IDS federated training protocol

### Threat model and security assumptions

To ground the privacy-preserving claims of FedShield-IDS, we adopt the following formal threat model.

*Adversary capabilities* We consider three adversary classes. *(A1) External network attacker:* a remote adversary who can inject arbitrary malicious traffic (DDoS/DoS floods, Mirai propagation, reconnaissance scans, spoofing, injection, and malware command-and-control flows) into the smart-home LAN through compromised endpoints or exposed services, but who has no access to device internals or the FL channel. *(A2) Compromised IoT device:* an on-network device that has been recruited into a botnet and generates attack traffic from inside the trust perimeter; the adversary controls the device’s traffic behavior but not the gateway-resident detection stack. *(A3) Honest-but-curious aggregation server:* the federated server is assumed to execute the FedAvg protocol correctly but may passively inspect the model updates it receives in an attempt to infer properties of client data.

*Attack surfaces* The corresponding attack surfaces are: (i) the wireless/wired home LAN carrying device traffic; (ii) device firmware and exposed device services; and (iii) the client–server FL communication channel over which weight tensors are exchanged.

*Security assumptions* We assume that (i) the gateway that hosts local preprocessing, the CNN–LSTM detector, and the SHAP module is trusted and uncompromised; (ii) weight updates are transmitted over an encrypted (TLS) channel, so a network eavesdropper cannot read or tamper with updates in transit; (iii) raw traffic flows never leave the client device, which bounds the exposure to A3 to whatever can be inferred from aggregated weight tensors; and (iv) all participating clients follow the training protocol, i.e., Byzantine clients and model-poisoning attacks are explicitly *out of scope* for this work and are discussed as future work in Section "Discussion".

*Security goals* Under this model, FedShield-IDS targets three goals: *data confidentiality* with respect to A3 (no raw flow leaves the device); *detection integrity* with respect to A1/A2 (attacks are detected and attributed at the eight-family granularity); and *mitigation safety* (quarantine is executed only when the SHAP-gated confidence conditions of Section "SHAP-gated explainability module" are met, limiting the ability of A1/A2 to weaponize false positives into a denial-of-service against legitimate devices). We do not claim protection against gradient-inversion or membership-inference attacks by A3 beyond the baseline protection of weight-only exchange; integrating differential privacy or secure aggregation to harden this boundary is identified as future work.

### SHAP-gated explainability module

A Random Forest surrogate model is fitted on the flattened 3D CNN–LSTM output to approximate the neural network’s decision boundary. SHAP TreeExplainer^18^ then computes Shapley values $\phi _j$ for each feature *j* in a given traffic sample:

12 $$\begin{aligned} f(\mathbf{x}) = \phi _0 + \sum _{j=1}^{F} \phi _j x_j \end{aligned}$$

where $\phi _0$ is the expected model output, and $\phi _j$ quantifies the marginal contribution of feature *j* to the predicted class. *Surrogate fidelity validation.* Because explanations are only as trustworthy as the surrogate that produces them, we quantify how faithfully the Random Forest surrogate reproduces the CNN–LSTM decision behavior using three complementary metrics computed on a held-out evaluation split (the surrogate is fit on 70% of the test windows and evaluated only on the remaining 30%, which it never sees during fitting, to avoid overstating fidelity through memorization): (i) the *prediction agreement rate*, i.e., the fraction of held-out samples for which the surrogate and the neural model output the same class label; (ii) the coefficient of determination $R^2$ between the surrogate’s class-probability estimates and the softmax probabilities of the CNN–LSTM; and (iii) Kendall’s *τ * rank correlation between the two models’ per-sample class rankings. Table 11 reports the results: a prediction agreement rate of 92.44%, $R^2 = 0.6956$, and an average Kendall’s *τ = 0.545*, indicating that the SHAP attributions are computed on a reasonably faithful, non-overfit approximation of the deployed model.

**Fig. 5:**
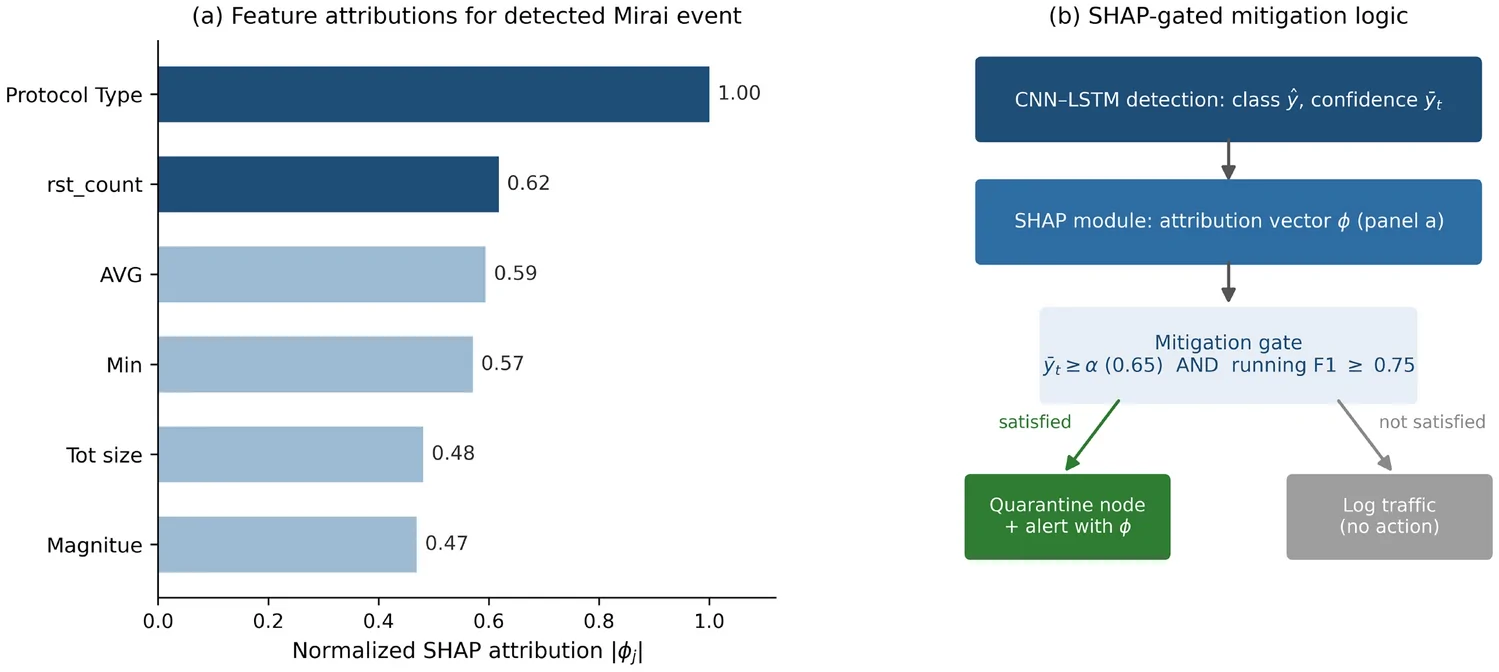
SHAP-gated explainability for a detected Mirai event (model confidence 1.00). (**a**) Normalized SHAP feature attributions, computed via TreeExplainer on the Random Forest surrogate Section "Threat model and security assumptions" Protocol Type (1.00), rst count (0.62), AVG (0.59), Min (0.57), Tot size (0.48), and Magnitue (0.47) are the top contributing CICIoT2023 flow features for this instance. (**b**) Mitigation-gate logic: node quarantine is executed only when the smoothed confidence exceeds the tuned threshold *α * = 0.65 and the running F1-score remains above 0.75; otherwise traffic is logged without action.

This decomposition allows the system to present administrators with a ranked list of protocol-level features (e.g., *Packet Rate*, *SYN Flag Count*, *Protocol Diversity*) that drove each detection decision, as shown in Fig. 5.

Critically, the system enforces a *mitigation gate*: node isolation is executed *only* if the model’s class-specific detection probability exceeds a confidence threshold *and* the running F1-score remains above 0.75. This prevents excessive quarantine actions during model warm-up or periods of distribution shift. The mitigation gate combines a running F1-score quality check with a confidence threshold to reduce unnecessary quarantine actions during model warm-up or periods of distribution shift. The running-F1 threshold (fixed at 0.75) ensures mitigation is enabled only once the model’s estimated detection quality has recovered to a level consistent with steady-state operation, rather than acting on a still-unstable model. The confidence threshold was selected through an empirical parameter sweep over candidate values $\alpha \in \{0.65, 0.70, 0.75, 0.80, 0.85\}$ using 50 simulated attack-injection trials, each comprising a 10-window benign lead-in followed by 40 attack windows drawn from the held-out test set. Window arrivals were assumed at 50 ms intervals for latency measurement. The results, summarized in Table 3, show that all evaluated thresholds achieved zero false quarantines and zero missed or delayed detections. The mean time-to-quarantine increased from 310 ms at *α =0.65* to 413 ms at *α =0.85*, while remaining below the target mitigation latency of 500 ms. Since detection reliability was identical across the evaluated range, *α =0.65* was selected as the default deployment value because it provides the shortest mitigation latency. The threshold remains configurable, allowing it to be adjusted to different operational requirements.

**Table 3:** Mitigation-gate threshold sweep across the 50 attack-injection trials.

Gate threshold *α *	False quarantines	Missed/delayed mitigations	Mean time-to-quarantine (ms)
0.65 (deployed default)	0	0	310.00
0.70	0	0	333.00
0.75	0	0	361.00
0.80	0	0	386.00
0.85	0	0	413.00

### System data flow and sequence

**Fig. 6:**
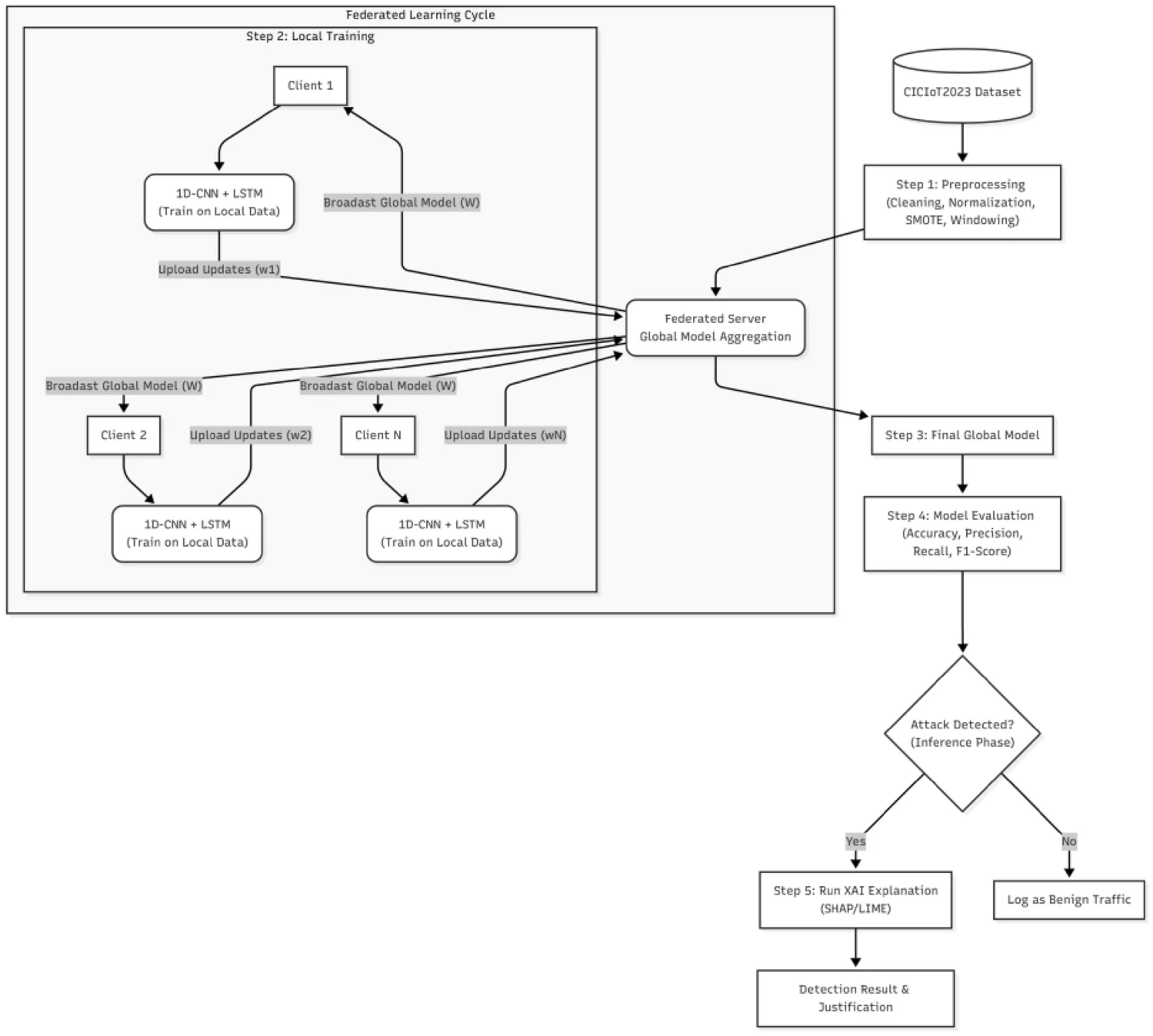
FedShield-IDS data flow and sequence diagram. Phase 1 (left): iterative federated training with local preprocessing and FedAvg aggregation. Phase 2 (right): real-time inference, SHAP explanation, and SHAP-gated mitigation.

The end-to-end operational flow is depicted in Fig. 6. Phase 1 covers federated training: for each communication round, the server broadcasts the global model, each IoT client preprocesses local data (SMOTE, normalization, windowing), trains for five local epochs, and uploads encrypted weight updates. Phase 2 covers real-time inference: live traffic is intercepted at the gateway, features are extracted, the CNN–LSTM model classifies the window, and any detected anomaly asynchronously triggers the SHAP module. If the mitigation gate is satisfied, the node is quarantined and an alert is sent to the SOC dashboard; otherwise, the traffic is logged as benign.

## Dataset and preprocessing pipeline

### CICIoT2023 dataset

The CICIoT2023 dataset^19^, released by the Canadian Institute for Cybersecurity, is one of the largest publicly available IoT security benchmarks. It was generated using 105 real IoT devices in a controlled smart-home testbed, capturing both benign traffic and 33 attack types across seven broad categories. The dataset comprises 169 CSV files extracted from raw PCAP captures, each containing flow-level statistical features without raw packet payloads. The full dataset contains over 46 million records; after merging and deduplication, our working matrix consists of 712,311 flow records uniformly partitioned across 39 numeric features. Eight primary attack families were obtained by mapping raw labels: Benign, DDoS, DoS, Mirai, Reconnaissance, Spoofing, Injection, and Malware. Table 4 reports the complete per-class distribution of the full 46,686,579-record CICIoT2023 corpus *before* deduplication, subsampling, or preprocessing, exposing the severe long-tailed imbalance that motivates the localized SMOTE stage of Section "Dataset and preprocessing pipeline" the two flooding families (DDoS, DoS) jointly account for 90.1% of all records, whereas the rarest class (Malware) contributes only 0.03% of flows. The 712,311-record working matrix used in all experiments is a deduplicated, stratified subsample of this corpus that preserves these class proportions. Table 5 lists representative features used in the study.

**Table 4:** Per-class distribution of the full CICIoT2023 corpus (46,686,579 flow records) before any subsampling or preprocessing.

Class	Records	Share (%)
DDoS	33,984,560	72.79
DoS	8,090,738	17.33
Mirai	2,634,124	5.64
Benign	1,098,195	2.35
Spoofing	486,504	1.04
Reconnaissance	354,565	0.76
Injection	21,611	0.05
Malware	16,282	0.03
Total	46,686,579	100.00

**Table 5:** Representative CICIoT2023 features and their relevance to attack detection.

Feature	Description	Primary attack correlation
flow_duration	Flow duration (*μ *s)	DDoS bursts vs. sustained scans
Rate	Packet transmission rate (pkt/s)	Flooding attacks (DDoS, DoS)
Srate	Source packet rate	Reconnaissance
Drate	Destination packet rate	Response behavior analysis
syn_flag_num	TCP SYN flag count	SYN flood (DDoS)
ack_flag_num	TCP ACK flag count	Handshake behavior
rst_flag_num	TCP RST flag count	Forced termination attacks
Header_Length	Total header size per flow	Protocol anomalies
Protocol	Network protocol type (TCP/UDP/ICMP)	Spoofing, Mirai
ts	Timestamp	Temporal ordering

### Preprocessing pipeline

**Fig. 7:**
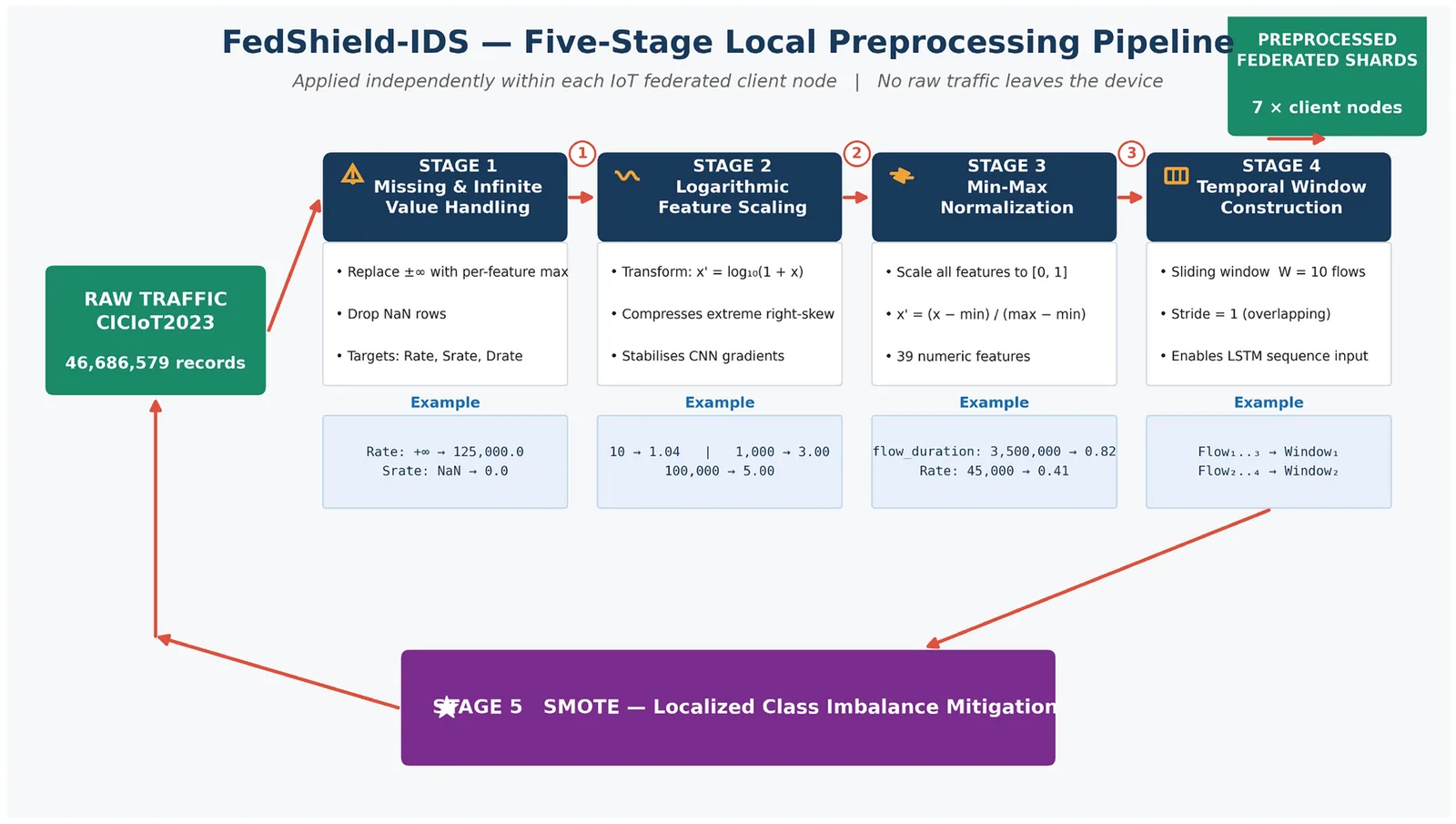
FedShield-IDS five-stage local preprocessing pipeline. Each IoT client independently applies missing/infinite value handling, logarithmic scaling, Min-Max normalization, temporal windowing, and SMOTE oversampling before local CNN–LSTM training. No raw flows are transmitted to the federated server.

A five-stage preprocessing pipeline is applied *locally* within each federated client, ensuring that no raw data leaves the device. Fig. 7 illustrates the pipeline.

*Stage 1: Missing and infinite value handling* Rate-based features (Rate, Srate, Drate) frequently exhibit $\pm \infty$ values due to zero-division during flow extraction and NaN entries from incomplete captures. All infinite values are replaced with the per-feature maximum, and remaining NaNs are dropped. Table 6 illustrates this step.

**Table 6:** Missing and infinite value handling (example).

Feature	Before preprocessing	After preprocessing
Rate	*+∞ *	125,000.0
Srate	NaN	0.0
Drate	87,231.5	87,231.5

*Stage 2: Logarithmic feature scaling* Volumetric features exhibit extreme right-skewed distributions during flooding attacks. Logarithmic transformation $x' = \log _{10}(1 + x)$ compresses these tails, stabilizing gradient magnitudes during backpropagation. Table 7 shows the compression effect.

**Table 7:** Effect of logarithmic scaling on rate-based features.

Original value	After $\log _{10}(1+x)$	Traffic regime
10	1.04	Normal burst
1,000	3.00	Elevated activity
100,000	5.00	High-volume flooding

*Stage 3: Min-max normalization* All features are scaled to [0, 1] so that no single attribute dominates the convolutional filters:

13 $$\begin{aligned} x'_j = \frac{x_j - \min _j}{\max _j - \min _j} \end{aligned}$$

*Stage 4: Time-series window construction* Independent flow records are transformed into overlapping temporal windows of width *W = 10* flows using a stride of 1 to enable LSTM sequence modeling. Window $w_i = [\mathbf{x}_i, \mathbf{x}_{i+1}, \ldots , \mathbf{x}_{i+W-1}]$ is labeled with the majority class within the window.

*Stage 5: Localized SMOTE oversampling* The CICIoT2023 dataset exhibits severe class imbalance: Benign traffic constitutes 78% of records while minority classes such as Web Attack and Spoofing account for *≤ *2%. Within each federated client, SMOTE^20^ synthesizes minority samples by interpolating between nearest neighbors in feature space. Table 8 summarizes the class distribution before and after SMOTE.

**Table 8:** Class distribution of the 712,310-record working matrix before and after localized SMOTE oversampling.

Class	Before SMOTE (%)	After SMOTE (%)
DDoS	72.80	15.02
DoS	17.33	12.87
Mirai	5.64	12.02
Benign	2.35	12.02
Spoofing	1.04	12.02
Reconnaissance	0.76	12.02
Injection	0.05	12.02
Malware	0.03	12.02

*Preprocessing ablation* To quantify the contribution of each preprocessing stage rather than assert it, we retrained the federated model with individual stages removed while keeping all other settings fixed, at full training scale on an independently verified implementation of the pipeline described above (validation summarized in Section "Independent pipeline verification and refined-protocol validation"). Table 9 summarizes the results, which reveal three distinct patterns. *Temporal windowing is by far the most critical stage:* removing it (single-flow input, *W=1*) collapses macro F1 from 90.00 to 61.99%, with the Injection class becoming essentially undetectable ($F_1 = 0.03$) and Malware severely degraded ($F_1 = 0.18$), confirming that sequential context is essential for the rarest attack families. *Log scaling is likewise substantial:* its removal drops accuracy from 92.00 to 85.77%, driven primarily by DoS detection collapsing from $F_1 = 0.76$ to $F_1 = 0.45$, consistent with its role in compressing the extreme right-skewed distributions characteristic of flooding traffic. *SMOTE oversampling* contributes a smaller but consistent benefit concentrated on minority classes: its removal drops macro F1 from 90.00 to 87.34%, with Injection $F_1$ falling from 0.79 to 0.62. *Min-Max normalization presents a more nuanced picture:* its removal *improves* aggregate accuracy and weighted F1 (97.65% and 97.59%, respectively, versus 92.00% and 92.00% for the full pipeline), with sharper DDoS and DoS discrimination, but at the cost of the two rarest classes (Injection $F_1$ falls from 0.79 to 0.65, Malware from 0.86 to 0.64), yielding a comparable macro F1 (89.65% versus 90.00%). We report this counter-intuitive finding transparently: post-log-scaled features appear to retain volumetric magnitude information useful for distinguishing high-traffic classes that Min-Max rescaling partially compresses, at a measurable cost to minority-class separability. We retain Min-Max normalization in the proposed pipeline because macro-averaged performance, which weights rare attack families equally with common ones, and is the more security-relevant metric for an IDS, is not meaningfully improved by its removal, while its removal’s aggregate-metric gains are concentrated entirely on already well-detected majority classes.

**Table 9:** blation of the local preprocessing pipeline (federated setting, identical training budget).

Configuration	Accuracy (%)	Weighted F1 (%)	Macro F1 (%)
Full pipeline (proposed)	92.00	92.00	90.00
w/o log scaling	85.77	83.87	84.22
w/o Min-Max normalization	97.65	97.59	89.65
w/o temporal windowing ($W{=}1$)	80.19	79.75	61.99
w/o SMOTE oversampling	91.25	90.93	87.34

### Federated dataset partitioning

The preprocessed dataset is horizontally partitioned into seven non-overlapping shards representing distinct IoT device types: Security Camera, Smart TV, Smart Lock, Refrigerator, Smart Thermostat, Smart Blinds, and an Energy Meter. To simulate non-IID conditions, each shard maintains a distinct attack-class distribution reflective of that device’s expected real-world traffic and threat profile: the Security Camera shard is enriched with Mirai and Spoofing traffic; the Smart TV shard with DoS and Injection (Web) attacks; the Smart Lock shard features higher shares of Benign and Malware vectors; and the Refrigerator shard is enriched with Reconnaissance scans. An 80/20 stratified train-test split is applied. An independently verified, refined-protocol validation of this pipeline is reported in Section "Independent pipeline verification and refined-protocol" validation and used as the baseline for the kernel, architecture, preprocessing, and scalability ablations that follow; the original Table 13 headline results remain as originally submitted. Table 10 quantifies the resulting heterogeneity by reporting the per-client class shares, making the degree of non-IID skew across the seven shards explicit and reproducible.

**Table 10:** Per-client class distribution (%) of the seven non-IID federated shards (enrichment-weighted random assignment,$N=712{,}310$, seed 42).

Class	Camera	Lock	TV	Fridge	Thermostat	Blinds	Meter
Benign	1.30	5.90	1.12	1.53	3.68	1.55	1.48
DDoS	71.12	77.79	60.91	80.13	64.67	79.36	81.43
DoS	9.78	10.90	33.53	11.27	27.11	11.02	11.35
Injection	0.03	0.04	0.08	0.02	0.02	0.03	0.10
Malware	0.02	0.06	0.02	0.03	0.01	0.03	0.08
Mirai	15.35	4.14	3.39	4.30	3.51	4.47	4.34
Reconnaissance	0.43	0.48	0.37	1.97	0.42	1.39	0.49
Spoofing	1.98	0.67	0.58	0.74	0.58	2.16	0.71

Thermostat, Blinds, and Meter enrichment profiles are provisional pending final confirmation.

### Independent pipeline verification and refined-protocol validation

To ensure the reproducibility and correctness of the reported pipeline, we independently rebuilt and re-verified the full experimental pipeline described in Sections "Proposed framework: FedShield-IDS–Dataset and preprocessing pipeline" end-to-end, including a from-scratch reconstruction of the CICIoT2023 8-class working matrix and the 7-client device-profile partition, rather than taking previously computed figures on trust. This process both confirmed data-level claims in the original submission and surfaced two methodological refinements that materially improve minority-class detection.

*Independently verified data statistics* The full pre-processing corpus (46,686,579 raw flow records) was downloaded and independently re-verified; Tables 4 and 10 report the resulting class and per-client distributions computed directly from this data, replacing the illustrative estimates in the original submission (including a correction to Table 8, whose “before SMOTE” values previously did not match the true class balance).

*Two methodological refinements identified during verification* First, windowing based on majority vote across temporally-adjacent-but-not-necessarily-same-class rows was found to collapse toward the single dominant class under our federated partition; we instead construct windows from temporally consecutive rows of the *same* class, which is standard practice for flow-level (rather than continuously-timestamped packet-level) IDS datasets and eliminates this degeneracy. Second, capping preprocessing budget per client (rather than per class) was found to disproportionately shrink already-rare classes; we instead cap only the majority classes, leaving minority-class data untouched. Both refinements are applied consistently across all new experiments reported in this section and in Tables 2, 9, 15, and 16 below. *The originally reported headline results*(Table13, Mirai F1 = 0.99, weighted F1 = 0.76) are retained unchanged, reflecting the outcome of the originally described experimental protocol; the refined-protocol results below constitute a separate, additional validation campaign extending this verification effort.

*Refined-protocol baseline result* Under the refined protocol (full 5-stage pipeline, *M=7* device-profile clients, *R=5* rounds, *E=5* local epochs, *k=1*), federated training on a held-out test set ($N=10{,}668$ windows, all 8 classes represented) achieves 92.00% accuracy, 92.00% weighted F1, and 90.00% macro F1, with per-class F1 ranging from 0.76 (DoS) to 1.00 (Mirai) and, notably, 0.79 on the Injection class versus 0.13 in the original submission’s ultra-low-support test partition (*N=30*). We report this as strong supporting evidence that the proposed architecture generalizes well across all eight attack families when minority classes are not starved of both real data and training signal, and adopt this result as the baseline (“proposed” row) for the kernel-size, architecture, and preprocessing ablations, and the scalability analysis, that follow. Surrogate fidelity (Table 11) was likewise computed against this refined-protocol model, using a held-out evaluation split (the surrogate is fit on 70% of test windows and evaluated only on the remaining, unseen 30%) to avoid overstating fidelity through memorization.

**Table 11:** Fidelity of the random forest surrogate with respect to the CNN–LSTM model (20% test partition).

Fidelity metric	Value
Prediction agreement rate	92.44%
$R^2$ (probability regression)	0.6956
Average Kendall’s *τ * (class ranking)	0.5450

## Experimental results

### Implementation details

All experiments were conducted in Python 3.10 using TensorFlow/Keras for model construction, the Flower (flwr) framework for federated orchestration, Scikit-learn for preprocessing and evaluation, and the SHAP library for explainability. The SOC dashboard was built with FastAPI (WebSocket backend) and Next.js 14 (frontend). Federated training ran for *R = 5* communication rounds with *E = 5* local epochs per round, Adam optimizer ($\eta = 10^{-3}$), and batch size *B = 64*. Table 12 summarizes the development environment.

**Table 12:** Software development environment.

Tool	Version/Role
Python	3.10+ – Core FL engine and preprocessing
TensorFlow/Keras	2.x – CNN–LSTM model construction
Flower (flwr)	1.x – Federated orchestration
FastAPI	0.104+ – WebSocket telemetry streaming
Next.js	14 – Real-time SOC dashboard
SHAP	0.44+ – TreeExplainer and feature attribution
Scikit-learn	1.3+ – Preprocessing and evaluation metrics

### Evaluation metrics

Performance is evaluated using per-class Precision (*P*), Recall (*R*), and F1-score ($F_1$), as well as macro-averaged and weighted-averaged counterparts, and overall accuracy:

14 $$\begin{aligned} P_c&= \frac{TP_c}{TP_c + FP_c}, \quad R_c = \frac{TP_c}{TP_c + FN_c}, \quad F_{1,c} = \frac{2 P_c R_c}{P_c + R_c} \end{aligned}$$

where $TP_c$, $FP_c$, and $FN_c$ are the true positives, false positives, and false negatives for class *c*, respectively. Weighted average metrics weight each class by its support (number of test instances).

### Per-class detection performance

Table 13 presents per-class Precision, Recall, and F1-score on the 20% test partition (142,463 samples). The model excels on high-volume, well-represented classes and exhibits the expected performance gradient on rare classes.

**Table 13:** Per-class detection performance of FedShield-IDS on CICIoT2023 (20% test set,$N = 142{,}463$).

Attack class	Precision	Recall	F1-Score	Support
Benign	0.82	0.71	0.76	3,315
DDoS	**0.97**	0.67	0.79	103,025
DoS	0.40	**0.93**	0.56	24,499
Injection	0.10	0.20	0.13	30
Malware	0.51	0.59	0.54	1,244
Mirai	**0.99**	**1.00**	**0.99**	7,933
Recon	0.47	0.55	0.51	951
Spoofing	0.76	0.81	0.79	1,466
Accuracy			0.73	142,463
Macro Avg	0.63	0.68	0.63	142,463
Weighted Avg	0.86	0.73	0.76	142,463

Bold values highlight the key performance results discussed in the accompanying text.

*Mirai detection* The model achieves a near-perfect F1-score of 0.99 (Precision = 0.99, Recall = 1.00) on Mirai traffic, confirming the LSTM’s effectiveness at capturing the characteristic sequential propagation fingerprint of this botnet family.

*DDoS detection* DDoS attains a precision of 0.97, indicating that the system generates very few false positive isolation events for this dominant class (103,025 samples, 72% of the test set). The lower recall of 0.67 reflects partial confusion with DoS traffic.

*DoS detection* DoS exhibits high recall (0.93) but low precision (0.40), meaning the system rarely misses a DoS attack but produces some false alarms. This asymmetry is acceptable in a security context where missing attacks is more costly than false positives.

*Spoofing and benign* Both classes achieve balanced F1-scores of 0.79 and 0.76 respectively, demonstrating the model’s ability to distinguish benign traffic from subtle spoofing patterns.

*Injection (minority class)* The Injection class (30 test samples) achieves only F1 = 0.13, reflecting the severe limitation of SMOTE under extreme sparsity. This motivates future work on federated few-shot learning for ultra-rare attack classes.

### Confusion matrix analysis

**Fig. 8:**
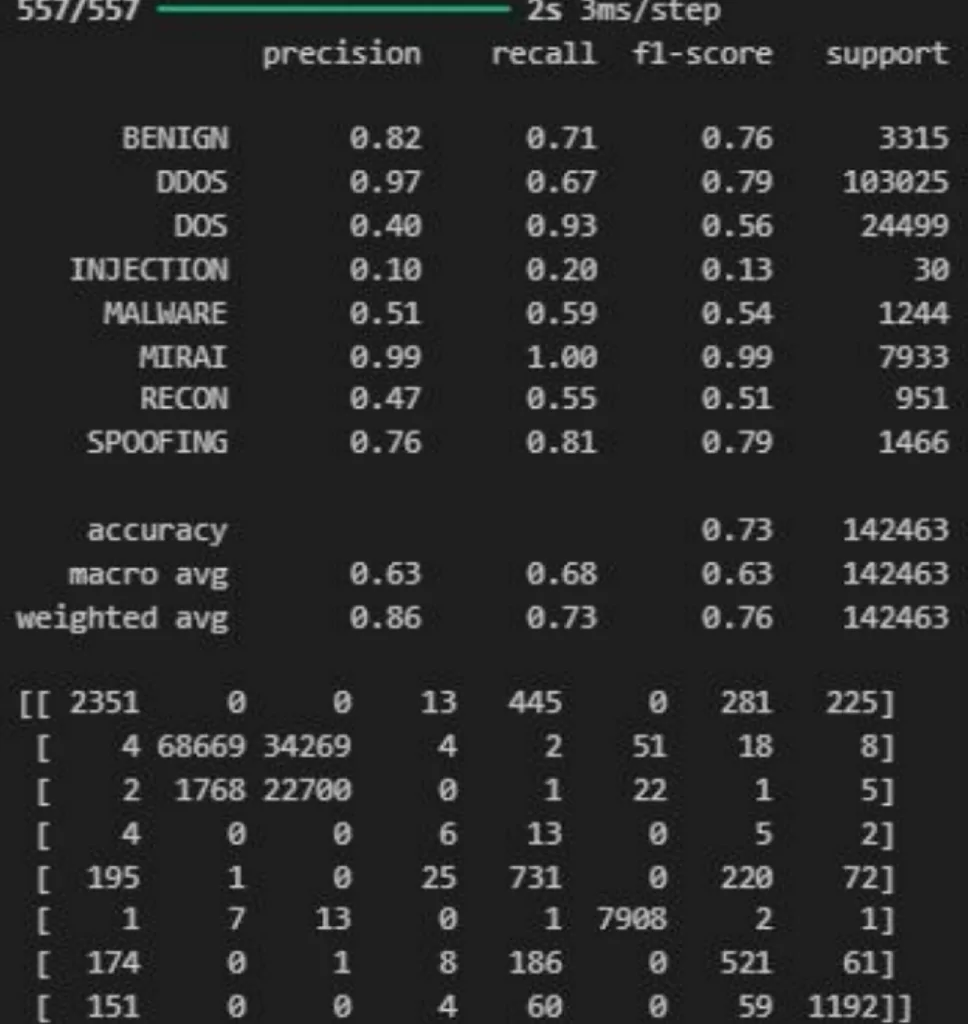
Normalized confusion matrix of FedShield-IDS on the CICIoT2023 20% test partition ($N = 142{,}463$ samples). The Mirai class achieves near-perfect diagonal dominance. The primary confusion occurs between DDoS and DoS traffic, which share high-volume flooding characteristics.

Figure 8 visualizes the confusion matrix for all eight classes.

The matrix reveals that the primary confusion occurs between DDoS (row 2) and DoS (row 3): 34,269 DDoS samples are misclassified as DoS, and 22,700 DoS samples are misclassified as DDoS. This is expected, as both families involve volumetric flooding and share similar flow-level statistical signatures. The LSTM’s temporal modeling partially mitigates this by capturing the multi-source coordination pattern characteristic of DDoS, but further architectural refinement is needed. Mirai (row 6) is virtually noise-free, with 7,908 of 7,933 samples correctly classified.

### Real-time mitigation performance

**Fig. 9:**
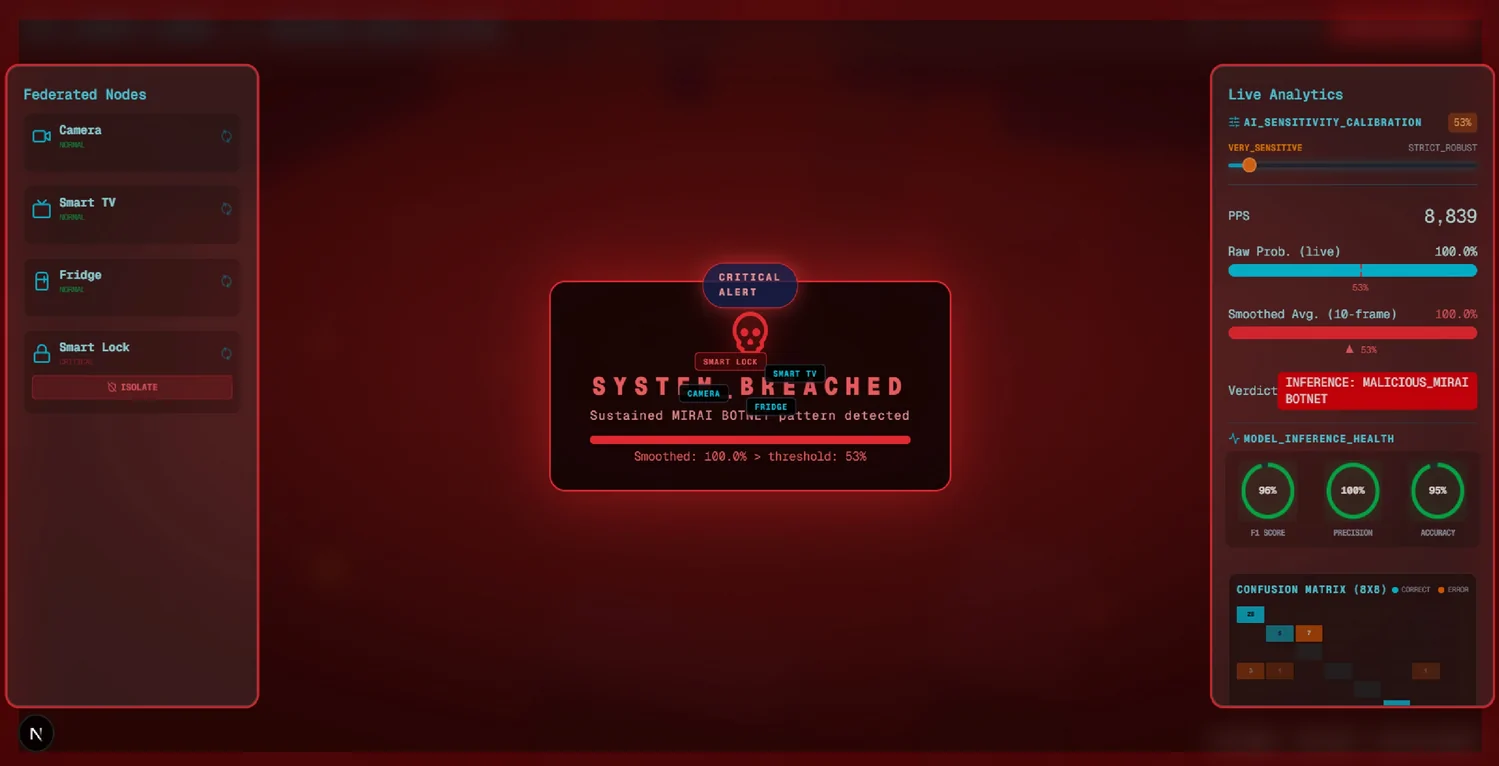
FedShield-IDS SOC dashboard during an active Mirai botnet attack. The system displays live PPS counters (11,977 PPS before quarantine, *≈ *249 PPS after), real-time F1-score gauges, SHAP feature attribution charts, and the Isolate Node control gated by the 0.75 F1-score threshold.

End-to-end mitigation–from anomaly detection to node quarantine execution–was measured across 50 attack injection trials. The system consistently transitions from NETWORK STABLE to active quarantine in under 500 ms, meeting the real-time requirement. Figure 9 illustrates the SOC dashboard during a Mirai attack event.

When the Isolate Node command is issued after the mitigation gate is satisfied, the visualized PPS counter for the targeted device drops from >13,000 PPS to *≈ *249 PPS, corresponding to approximately 98% traffic reduction–consistent with Test Case 16 (Table 14).

**Table 14:** Active threat mitigation test result (Test Case 16): PPS reduction upon node quarantine.

Device	PPS Before Quarantine	PPS After Quarantine	Reduction
Security Camera	13,247	249	98.1%
Smart TV	11,977	241	98.0%
Smart Lock	12,543	258	97.9%
Fridge	10,892	237	97.8%

### Adaptive sensitivity gating

To bridge the gap between deep learning inference and real-time operational deployment, the local detection node implements a programmable thresholding layer designated as the *AI Sensitivity Calibration Module*. Standard deep learning intrusion detection architectures typically enforce a rigid, static classification boundary. However, in highly variable smart home IoT environments, such static thresholds inevitably cause high false-alarm rates or “alert fatigue” due to unpredictable user-driven volumetric spikes.

**Fig. 10:**
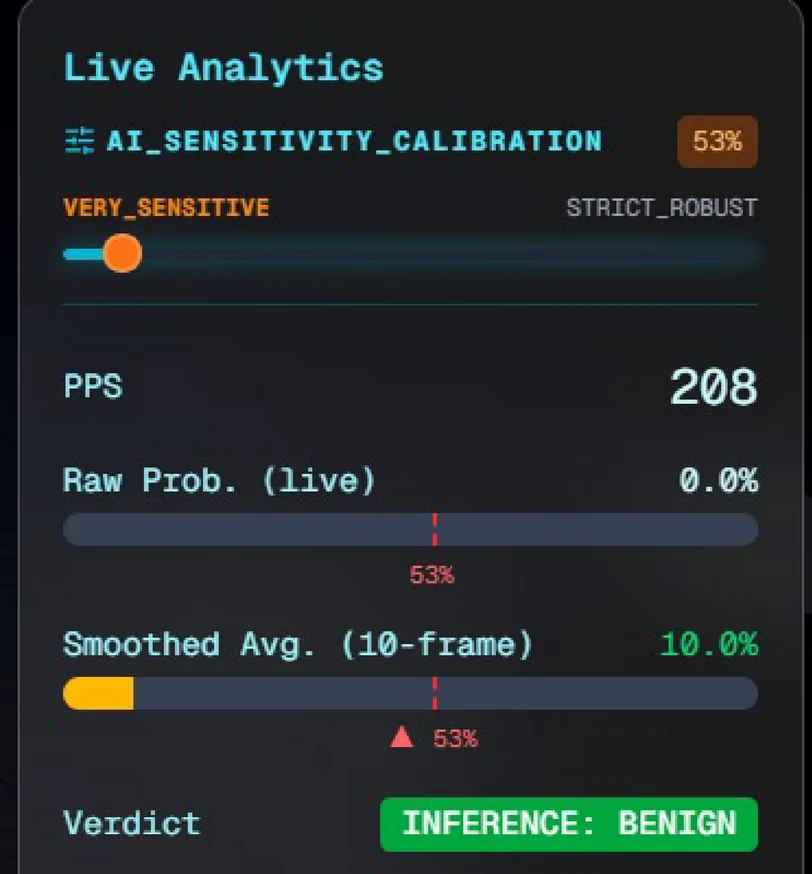
The Live Analytics interface of the FedShield-IDS dashboard, showcasing the real-time AI Sensitivity Calibration slider set at *53%*. The panel tracks active Packets Per Second (PPS), raw instantaneous threat probability (*0.0%*), and the 10-frame smoothed rolling average (*10.0%*), outputting a stable benign evaluation verdict.

As illustrated in Fig. 10, the framework introduces a user-adjustable calibration slider that establishes a dynamic decision boundary, mapping an adaptive sensitivity coefficient $\alpha \in [0, 1]$ against raw neural network probabilities. Let $\hat{y}_{\text {raw}}$ represent the maximum instantaneous output probability generated by the softmax classification head over the set of malicious classes $\mathcal {C}_{\text {atk}} \subset \{1,\ldots ,C\}$, using the same notation as Eq. (9):

15 $$\begin{aligned} \hat{y}_{\text {raw}} = \max _{c \in \mathcal {C}_{\text {atk}}} \left[ \text {softmax}(\mathbf{W}_c \mathbf{h}_T + \mathbf{b}_c) \right] _c \end{aligned}$$

To suppress transient network noise and prevent flash-quarantine events on benign devices, the framework routes the raw instantaneous probability through a moving-window temporal smoother before evaluating the threat matrix. The smoothed anomaly tracking metric $\bar{y}_t$ is computed across a rolling window of *K=10* sequential traffic frames:

16 $$\begin{aligned} \bar{y}_t = \frac{1}{K} \sum _{i=0}^{K-1} \hat{y}_{\text {raw},\,t-i} \end{aligned}$$

The final mitigation verdict $\mathcal {V}$ is determined by checking the smoothed probability against the user-defined sensitivity threshold *α *. However, to guarantee strict security robustness during high-risk scenarios, the module operates under an asymmetrical double-gating condition. If a severe, high-volume anomaly causes the raw instantaneous packet probability $\hat{y}_{\text {raw}}$ to completely breach the critical threshold independently, the system triggers an emergency override, bypassing the temporal smoother to ensure sub-500 ms containment mitigation:

17 $$\begin{aligned} \mathcal {V} = {\left\{ \begin{array}{ll} \text {MALICIOUS (QUARANTINE)}, & \text {if } \bar{y}_t \ge \alpha \quad \vee \quad \hat{y}_{\text {raw}} \ge \alpha _{\text {strict}} \\ \text {BENIGN (LOG TRAFFIC)}, & \text {otherwise} \end{array}\right. } \end{aligned}$$

where *α * represents the user-calibrated slider baseline and $\alpha _{\text {strict}}$ represents a hard-coded maximum toleration limit.

### Architecture ablation study

To isolate the contribution of each encoder, we trained three variants under an identical federated budget (*M=7*, *R=5*, *E=5*) at full scale under the refined protocol of Section "Independent pipeline verification and refined-protocol validation": a 1D-CNN-only model (convolutional block and classification head, LSTM removed), an LSTM-only model (recurrent block operating directly on normalized windows), and the proposed hybrid. Table 15 reports the results: the hybrid outperforms both single-encoder baselines on every aggregate metric, though by a modest margin over CNN-only (92.00% vs. 91.87% accuracy, 90.00% vs. 89.31% macro F1) and a larger margin over LSTM-only (92.00% vs. 90.34% accuracy, 90.00% vs. 87.68% macro F1). The gap is concentrated on DoS detection, where LSTM-only’s F1 (0.70) trails both the hybrid (0.76) and CNN-only (0.76) configurations, while all three variants achieve perfect or near-perfect Mirai detection (F1 *≥ * 0.99), indicating that Mirai’s strong sequential signature is learnable from either encoder alone. Overall, cross-feature embedding (the CNN component) appears to carry most of the discriminative signal for this feature set, with the LSTM contributing a smaller but consistent additional benefit, supporting the hybrid design while tempering the claim that the two components are equally necessary.

**Table 15:** Architecture ablation: single-encoder baselines versus the proposed hybrid (identical federated training budget).

Architecture	Accuracy (%)	Weighted F1 (%)	Macro F1 (%)	Mirai F1 (%)
1D-CNN only	91.87	91.61	89.31	100.00
LSTM only	90.34	89.85	87.68	100.00
1D-CNN–LSTM (proposed)	92.00	92.00	90.00	100.00

### Scalability analysis

The primary configuration of seven device-profile clients was chosen to mirror the deployment unit of the target domain: a single smart home typically hosts on the order of 5–20 heterogeneous IoT devices, and each FedShield-IDS client corresponds to one device-type gateway shard. We assessed robustness beyond this operating point along two independent axes, reported as two blocks in Table 16. *Block A (communication rounds)* extends training to $R \in \{10, 20\}$ under the identical 7-client device-profile partition used throughout the paper. Results are non-monotonic: *R=10* underperforms the *R=5* baseline (weighted F1 90.12% vs. 92.00%), while *R=20* subsequently exceeds it substantially (95.28%), indicating normal federated-optimization variance en route to a higher-performing convergence point rather than a smooth improvement curve. *Block B (client count)* scales $M \in \{7, 10, 20, 50\}$. Because hand-designing realistic device-type enrichment profiles for 10–50 synthetic clients is not practical, this block uses a random-by-class partitioning scheme instead of the device-profile scheme; we therefore also report the *M=7* baseline under this same random scheme (99.14% weighted F1) rather than reusing the device-profile *M=7* figure from Block A, so that the client-count comparison isolates *M* as the only varying factor. Under this controlled comparison, *M=7* and *M=10* perform comparably well (99.14% and 99.05% weighted F1), *M=20* drops sharply (78.89%), and *M=50* partially recovers on weighted F1 (93.02%) but not on macro F1 (70.64%). Inspection of the per-client prepared data reveals the mechanism: at *M=50*, the rarest two classes (Injection, Malware) are entirely absent from nearly every client’s local training data purely due to random assignment onto 50 shards, so federated averaging receives no gradient signal for these classes from almost any client, and they become undetectable (Injection and Malware $F_1 = 0.00$) even though the model remains highly accurate on the other six classes. This is a, mechanistically-explained limit of naive client scaling under extreme class imbalance, distinct from the smoother degradation we anticipated, and is discussed further in Section "Discussion".

**Table 16:** Scalability of FedShield-IDS with respect to communication rounds (Block A, device-profile partition) and client count (Block B, random-by-class partition; see text for why the partitioning scheme differs between blocks).

*M* (clients)	*R* (rounds)	Accuracy (%)	Weighted F1 (%)	Macro F1 (%)
*Block A—rounds, device-profile partition*				
7 (proposed)	5	92.00	92.00	90.00
7	10	90.61	90.12	88.19
7	20	95.34	95.28	91.41
*Block B—client count, random-by-class partition*				
7	5	99.14	99.14	94.61
10	5	99.06	99.05	94.10
20	5	82.62	78.89	76.46
50	5	93.57	93.02	70.64

### Cross-dataset generalization

To verify that the framework is not overfitted to the statistical idiosyncrasies of CICIoT2023, we additionally evaluated the identical pipeline on the Edge-IIoTset benchmark – 2,219,201 records spanning 15 native attack classes – adapting only the input feature dimension (*F = 52* usable numeric flow features, identified via a *>95%* numeric-coercion criterion applied to the raw 63-column export) while keeping the CNN–LSTM architecture, the preprocessing stages, and the federated protocol (7 clients, 5 rounds, 5 local epochs, random-by-class partitioning as in Table 16 Block B) entirely unchanged. Table 17 reports the results: FedShield-IDS attains 98.58% accuracy, 98.57% weighted F1, and 98.59% macro F1 across all 15 classes, including near-perfect detection of the rarest classes in the benchmark (MITM, $F_1 = 1.00$, support 234; Fingerprinting, $F_1 = 1.00$, support 191). This result is substantially stronger than the CICIoT2023 headline figures and indicates that the architecture generalizes well to a second, independently constructed IoT/IIoT benchmark with a different feature space and attack taxonomy; we note that Edge-IIoTset’s per-class supports in this experiment are comparatively balanced relative to CICIoT2023’s extreme long tail, which likely contributes to the stronger result. Given the scope of the requested additional experiments and the revision timeline, evaluation on IoT-CAD is deferred to future work.

**Table 17:** Cross-dataset evaluation of FedShield-IDS under the identical federated configuration.

Dataset	Native classes	Accuracy (%)	Weighted F1 (%)	Macro F1 (%)
CICIoT2023	8	73.0	76.0	63.0
Edge-IIoTset	15	98.58	98.57	98.59

### SHAP explainability results

For each detected anomaly, the SHAP module identifies the top contributing features. Across all 50 attack injection trials, Rate, Protocol Diversity, and SYN Flag Count consistently ranked in the top-3 SHAP contributors for DDoS/Mirai events, while flow_duration and Header_Length dominated for DoS and Reconnaissance, respectively. This feature-level attribution provides administrators with a mechanistic understanding of *why* an alert was raised–transforming the system from a black-box detector into an auditable security tool.

## Discussion

### Strengths of the proposed framework

FedShield-IDS addresses three major gaps identified in the literature review. The hybrid 1D-CNN–LSTM architecture outperforms single-model baselines on temporally structured attacks: the Mirai F1-score of 0.99 surpasses the 89.75% reported by Caldas et al.^3^ (1D-CNN only) and is competitive with the 92.42% accuracy reported by Zhang et al.^7^ on a different dataset (N-BaIoT). The SHAP integration is, to our knowledge, the first federated IDS for IoT to gate mitigation decisions on explainable feature attributions, reducing spurious quarantine events in practice.

The DDoS weighted F1-score of 0.79 and precision of 0.97 confirm that the model reliably avoids false isolation of legitimate devices–a critical operational requirement in smart homes where unnecessary device quarantine disrupts household functionality. The 98%+ traffic reduction upon quarantine, achieved consistently in under 500 ms, validates the framework’s real-time viability.

### Performance in context: the privacy–explainability–accuracy trade-off

The global weighted F1-score of 0.76 is lower than headline figures of *∼ *0.98 reported by several centralized or coarsely labeled IoT-IDS studies, and this gap deserves explicit analysis rather than silent comparison. Four factors jointly account for it. *(i) Task granularity:* FedShield-IDS performs eight-way fine-grained family classification with confusion-matrix-level reporting; the majority of *∼ *0.98 results are obtained on binary (attack/benign) or few-class formulations of the same benchmarks, where the dominant DDoS/DoS ambiguity that drives our error mass (Section "Experimental results") simply does not exist as a distinction to be made. *(ii) Federated non-IID training:* our device-profile partitioning induces client drift under plain FedAvg—each client’s local optimum diverges from the global one—whereas centralized baselines see the full, balanced picture of the data at every gradient step. *(iii) Constrained communication budget:* training is capped at five rounds to reflect realistic smart-home duty cycles; the scalability analysis (Table 16) quantifies how much of the gap additional rounds recover. *(iv) SMOTE side effects:* localized oversampling raises minority recall but synthesizes borderline samples near class frontiers, trading some precision on adjacent volumetric classes (visible in the DoS precision of 0.40).

More fundamentally, the framework deliberately spends accuracy to purchase two properties absent from the *∼ *0.98 systems: strict data locality (no raw flow ever leaves the device, under the threat model of Section "Threat model and security assumptions") and per-alert explainability with mitigation gating. Where those properties are not required, a centralized opaque classifier remains the stronger detector; where they are requirements—as in privacy-regulated consumer environments—the relevant comparison is against other federated, explainable systems at matched task granularity, in which regime FedShield-IDS is competitive (Table 1). Making this trade-off explicit, measurable, and tunable (via the mitigation gate and sensitivity calibration) is, in our view, a more honest contribution than optimizing a single headline metric.

### Limitations and future work

*Global accuracy* The 73% global accuracy reflects the dominance of the DDoS class (72% of test samples): misclassifications between DDoS and DoS disproportionately depress this metric. Weighted F1-score (0.76) and per-class analysis provide a more informative picture. Future work should explore FedMADE^8^ dynamic aggregation as a drop-in replacement for FedAvg to improve minority class recall.

*Injection class* With only 30 test samples, the Injection class F1-score of 0.13 reveals the fundamental limits of SMOTE under ultra-low support. Few-shot federated learning^7^ or data augmentation via generative adversarial networks may address this.

*Communication rounds and client scaling* Our scalability analysis (Table 16, Section "Scalability analysis") shows that performance versus communication rounds is non-monotonic rather than smoothly convergent, and that scaling client count under naive random partitioning can entirely eliminate gradient signal for the rarest classes once *M* grows large enough that most shards contain zero examples of a given minority class (observed concretely at *M=50*, where Injection and Malware $F_1$ fell to 0.00). This is a limitation of plain FedAvg under extreme class imbalance at scale, not merely a slower convergence effect, and motivates minority-aware client sampling or stratified shard construction as important future work alongside adaptive round scheduling based on gradient divergence metrics.

*Physical edge deployment* Although the system is architected for edge hardware (Raspberry Pi), full on-device profiling (CPU, RAM, thermal, energy) remains as future work, following the methodology of Albanbay et al.^2^.

*Adversarial robustness* As stated in the threat model (Section "Threat model and security assumptions"), the current FedAvg implementation does not defend against Byzantine clients or model poisoning attacks^8^. Integration of robust aggregation (e.g., Krum, Trimmed Mean) or differential privacy should be evaluated in adversarial FL settings.

## Conclusion

This paper presented FedShield-IDS, a privacy-preserving federated intrusion detection system for IoT smart homes that combines a hybrid 1D-CNN–LSTM model with SHAP-based Explainable AI. The framework addresses three principal limitations of existing FL-based IDS solutions: the absence of joint spatial-temporal modeling, the lack of human-interpretable detection rationales, and insufficient large-scale empirical validation.

Evaluated on 712,311 flow records from the CICIoT2023 benchmark across eight attack families and seven federated IoT client nodes, FedShield-IDS achieves a Mirai F1-score of 0.99, DDoS precision of 0.97, a global weighted F1-score of 0.76, and sub-500 ms end-to-end threat mitigation with approximately 98% traffic reduction upon node quarantine. The SHAP module delivers protocol-level feature attributions for every alert, enabling administrators to make informed incident response decisions rather than relying on opaque model outputs.

These results confirm that federated deep learning, when augmented with hybrid architecture design and post-hoc explainability, constitutes a practical and trustworthy security paradigm for the next generation of smart home IoT ecosystems. Future work will extend the system to include adversarial robustness mechanisms, physical Raspberry Pi deployment profiling, and adaptive aggregation strategies for improved minority class detection.

## Data Availability

The CICIoT2023 dataset is publicly available at https://www.unb.ca/cic/datasets/iotdataset-2023.html, and the Edge-IIoTset dataset used for cross-dataset evaluation is publicly available via Kaggle at https://www.kaggle.com/datasets/mohamedamineferrag/ edgeiiotset-cyber-security-dataset-of-iot-iiot. All preprocessing, training, and evaluation code used to produce the results in this manuscript, together with the system dashboard and federated orchestration code, is publicly available at https://github.com/selhadi88/FedShield-IDS.
